# Acquisition and Analysis of DIA-Based Proteomic Data: A Comprehensive Survey in 2023

**DOI:** 10.1016/j.mcpro.2024.100712

**Published:** 2024-01-03

**Authors:** Ronghui Lou, Wenqing Shui

**Affiliations:** 1 iHuman Institute, ShanghaiTech University, Shanghai, China; 2 School of Life Science and Technology, ShanghaiTech University, Shanghai, China

**Keywords:** bottom-up proteomics, data-independent acquisition, DIA data analysis, spectral library, benchmark data

## Abstract

Data-independent acquisition (DIA) mass spectrometry (MS) has emerged as a powerful technology for high-throughput, accurate, and reproducible quantitative proteomics. This review provides a comprehensive overview of recent advances in both the experimental and computational methods for DIA proteomics, from data acquisition schemes to analysis strategies and software tools. DIA acquisition schemes are categorized based on the design of precursor isolation windows, highlighting wide-window, overlapping-window, narrow-window, scanning quadrupole-based, and parallel accumulation-serial fragmentation–enhanced DIA methods. For DIA data analysis, major strategies are classified into spectrum reconstruction, sequence-based search, library-based search, *de novo* sequencing, and sequencing-independent approaches. A wide array of software tools implementing these strategies are reviewed, with details on their overall workflows and scoring approaches at different steps. The generation and optimization of spectral libraries, which are critical resources for DIA analysis, are also discussed. Publicly available benchmark datasets covering global proteomics and phosphoproteomics are summarized to facilitate performance evaluation of various software tools and analysis workflows. Continued advances and synergistic developments of versatile components in DIA workflows are expected to further enhance the power of DIA-based proteomics.

## Highlights

• Comprehensive overview of various DIA acquisition schemes in three major classes.
• Classification and overview of strategies and software tools for DIA data analysis.
• Introduction of the generation of spectral libraries in diverse DIA workflows.
• Summary of published benchmark datasets for evaluation of DIA acquisition methods and workflows.

## Graphical Abstract

**Figure:**
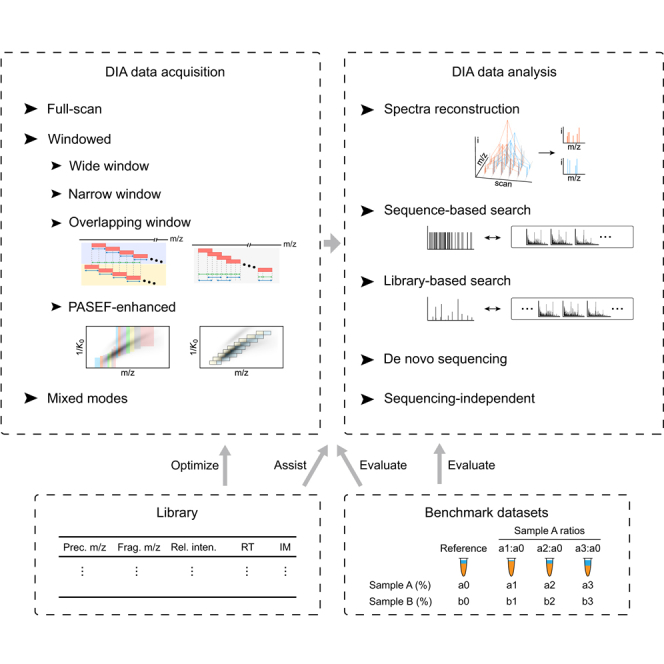

## In Brief

In this review, we comprehensively categorize and describe data-independent acquisition schemes and associated software tools, primarily in a chronological order. Our review also covers the major roles of spectral libraries and useful benchmark datasets. A notable feature is the systematic classification of acquisition schemes and data analysis tools, enhancing our understanding of historical and recent advances in DIA-based proteomics.

Mass spectrometry (MS)-based bottom-up proteomics has become one of the most powerful technologies for large-scale profiling of the proteome composition and dynamic regulation in diverse biological systems and clinical specimens (1, 2, 3). Owing to significant advances in both MS instruments and informatic pipelines, current bottom-up proteomics has attained a coverage of the expressed protein-coded genes at a depth comparable to transcriptomics yet provided additional insights into protein posttranslational modification and protein complex assembly (4, 5, 6, 7, 8).

In discovery-oriented bottom-up proteomics, two widely adopted data acquisition strategies, namely data-dependent acquisition (DDA) and data-independent acquisition (DIA), mainly differ in the way of isolating precursor ions for fragmentation and subsequent MS2 spectra acquisition. Briefly, a DDA experimental scheme typically comprises the selection, accumulation, and fragmentation of precursor ions based on real-time analysis of data/signals from an MS1 survey scan. In contrast, the mass spectrometer in DIA experiments cycles through a predefined set of precursor isolation windows within which all the precursor ions are simultaneously fragmented, obviating the need for real-time precursor selection. However, compared to DDA, DIA usually generates inherently complex MS2 spectra and multiplexed chromatograms with reduced precursor selectivity, thus requiring specialized informatic tools for DIA data analysis. Over the last 2 decades, a wide variety of DIA data acquisition schemes have been proposed and implemented on different types of MS instrument platforms, continuously pushing the boundaries of sensitivity, specificity, reproducibility, and throughput achievable by DIA (9, 10, 11, 12, 13, 14). The data acquisition advancement has been accompanied by the development of diverse data analysis strategies and software tools to effectively decipher the original DIA ion map.

The experimental workflow, informatic tools, and biological applications of DIA-based proteomics have been outlined in previous reviews with different emphasis (9, 10, 11, 12, 13, 15, 16). In this review, we provide a comprehensive and updated overview of DIA data acquisition schemes, strategies and software tools for DIA data analysis, and benchmark datasets for workflow evaluation. Compared to previous literature, this review particularly focuses on the evolution and recent advances of the acquisition and analysis of DIA-based proteomic data for which various methods are classified into distinct categories. Alongside this review, an online appendix with much more details is provided on the GitHub page (https://Shui-Group.github.io/DIAReviewAppendix).

## DIA Data Acquisition Schemes

**Fig. 1:**
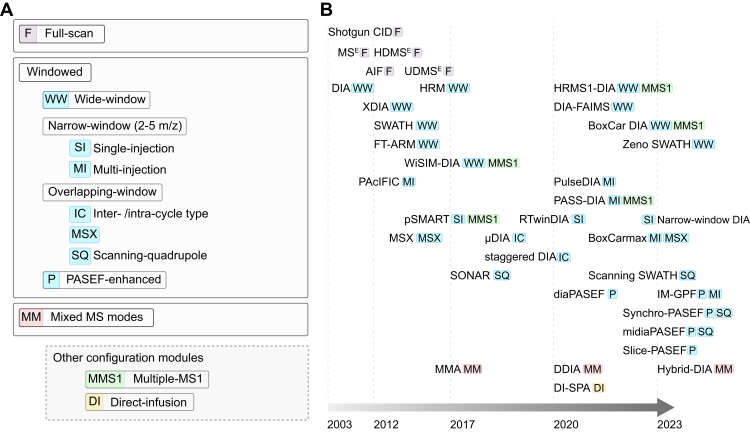
**Categoriesof DIA acquisition schemes and their development timeline.***A*, DIA acquisition schemes are classified into three main categories: full-scan, windowed, and mixed MS modes. Windowed DIA is further divided based on the size and arrangement of isolation windows. Other configurations can be incorporated into existing schemes. *B*, timeline showing the development of various DIA acquisition schemes.

Depending on the design of precursor isolation windows, we classify all reported DIA data acquisition schemes into three major categories: full-scan DIA, windowed DIA, and unconventional methods (Fig. 1*A*). Being the most widely employed scheme in current DIA-based proteomic studies, windowed DIA is subdivided into wide-window, narrow-window, overlapping-window, scanning quadrupole-based, and parallel accumulation-serial fragmentation (PASEF)-enhanced DIA. The category of unconventional methods includes mixed-mode DIA and direct-infusion DIA. In addition to the variation in the MS2 window design and instrument configuration, the MS1-enhanced method is a special category of acquisition setting and in principle it can be combined with any of the aforementioned DIA schemes. Specific DIA methods falling into different categories are annotated alongside the timeline of method development (Fig. 1*B*).

### Full-Scan DIA

**Fig. 2:**
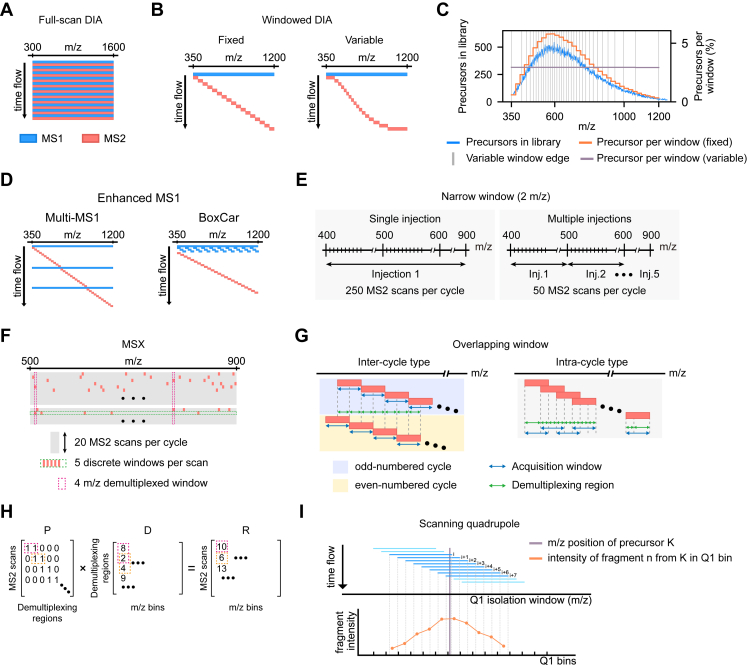
**Overview of DIA acquisition schemes.***A*, full-scan DIA isolates all precursors across the entire m/z range for a single MS2 scan per cycle. *B*, windowed DIA uses fixed or variable isolation window sizes to cover the m/z range with multiple MS2 scans per cycle. *C*, using 34 fixed windows results in uneven distribution of precursors in the Pan Human library (24) across windows (*orange line*) mirroring the precursor distribution in the library (*blue line*). Variable windows (*gray vertical lines*) enable nearly even precursor distributions (*purple line*). *D*, MS1-enhanced methods insert multiple full-range or segmented MS1 scans per cycle. *E*, single-injection narrow-window DIA requires extensive MS2 scans (*e.g.* 250 for a 2 m/z window over the 400–900 m/z range) while multi-injection reduces them to 50 scans per cycle (five injections shown here). *F*, in MSX, each of 20 MS2 scans contains 5 m/z segments, enabling 5-fold demultiplexing. *G*, overlapping windows interleave between cycles (inter-cycle type) or within a cycle (intra-cycle type). *H*, demultiplexing algorithm introduced with MSX (53). For inter-cycle overlapping DIA, the presence matrix (P) comprises rows of MS2 scans and columns of demultiplexing regions, where entries are one for a region aligned to a scan, otherwise 0. P multiplies the solving matrix (*D*) of region intensities to yield the concatenated binned intensity matrix (R). In D, rows are demultiplexing regions and columns are m/z bins. In R, rows are real scans and columns are m/z bins. *D* is then solved by non-negative least-squares. *I*, in scanning quadrupole DIA, a precursor is covered by multiple Q1 isolation windows (i to i + 7), generating a Q1 profile for each fragment by summing spectra falling within each Q1 bin.

The core principle shared by full-scan DIA methods is isolating precursor ions across the entire expected m/z range, such as 300 to 1600 m/z, and executing a single MS2 scan per cycle where all accumulated precursors are fragmented (Fig. 2*A*).

The full-scan DIA techniques include Shotgun-collision-induced dissociation (CID) (17), MS^E^ (18), all-ion fragmentation (19), HDMS^E^ (20), and UDMS^E^ (21). Shotgun-CID was developed in 2003 on a time-of-flight (TOF) instrument with an electrospray ionization source, using nozzle-skimmer CID for in-source fragmentation. Two runs were acquired for each sample, with one using a fixed low voltage to sample unfragmented precursor ions and another using a fixed high voltage to sample fragmented ions. Subsequently, MS^E^ and all-ion fragmentation emerged in 2005 and 2011, on Waters Synapt and Thermo Exactive instruments, respectively. They utilized in-cell fragmentation with switchable collision energies to acquire interleaved MS1 and MS2 scans within a single run.

In 2006, Waters introduced traveling wave ion mobility spectrometry to the Synapt HDMS instrument (22), named as HDMS^E^ which incorporates additional mobility separation compared to MS^E^ (20). Building on hardware enhancements, Ute *et al.* implemented UDMS^E^ on the Synapt G2-S instrument in 2014, using drift time-dependent collision energies to address under- or over-fragmentation resulting from the constant collision energy used in HDMS^E^ (21, 23).

### Wide-Window DIA

Due to the low specificity in precursor selection, full-scan DIA generally yields highly complex MS2 spectra, limiting its data interpretation. To lower interference signals from overlapping precursors and fragments, windowed DIA methods are proposed to partition the full m/z range into segments allocated to multiple MS2 scans per cycle (Fig. 2*B*). Using the precursor distribution in the Pan Human library (24) as an illustration, covering the 350 to 1200 m/z range with 34 fixed windows is expected to reduce spectral complexity in each window (Fig. 2*C*). However, such a design does not solve the issue of uneven precursor distribution. In comparison, variable window division, typically inferred from a spectral library or total ion currents, enables the concurrent management of a more comparable number of precursor species between windows (25) (Fig. 2, *B* and *C*).

Among windowed DIA methods, wide-window DIA is the most common, owing to its easy configuration and broad compatibility with various MS instruments. In the original DIA blueprint proposed by Venable *et al.* in 2004 (26), a 10 m/z window size was used to cover the 400 to 1400 m/z range, yielding 100 MS2 scans and approximately a 35 s cycle time on an LTQ instrument. Then specialized window designs have been proposed in a number of methods tailored to specific instruments. For example, XDIA (extended DIA), introduced by Carvalho *et al.* in 2010 (27), used a 20 m/z window to cover the 400 to 1000 m/z range, with 60 MS2 scans per cycle. Each isolation window was scanned twice with ion dissociation executed through electron transfer dissociation (ETD) or ETD followed by CID on the LTQ-Orbitrap XL instrument.

In 2012, FT-ARM (Fourier transform-all reaction monitoring) (28) and SWATH(-MS) (sequential window acquisition of all theoretical (mass spectra)) (29) methods were developed for the LTQ-FT or LTQ Orbitrap and TripleTOF 5600 instruments, respectively. FT-ARM capitalizes on high-resolution instruments to produce high resolution/accurate mass (HR/AM) MS2 spectra, using a 100 m/z isolation window to cover the 500 to 1000 m/z range. In contrast, SWATH segments the broader 400 to 1200 m/z range into 25 m/z slices. Moreover, SWATH collision energies can be optimized in a finely tuned manner for each window. More importantly, this pioneering work of SWATH-MS, established the spectral library-based strategy for MS2 spectra deconvolution. In 2015, Biognosys introduced HRM (hyper reaction monitoring) to the Orbitrap instrument family, which combines high-resolution MS2 scans with variable window segmentation while maintaining a favorable cycle time (30).

Additional methods tailored to specific MS instruments, such as DIA-FAIMS (31), Zeno SWATH (32), and diaPASEF (33), also offer advantages. DIA-FAIMS operates on quadrupole-FAIMS-MS instruments like Thermo Exploris 480, which leverages differential ion mobility spectrometry (IMS) for additional precursor ion separation. Thus this acquisition scheme allows enhanced dynamic range, higher sensitivity, and cleaner MS2 spectra. The Zeno SWATH technique was developed based on an extra linear ion trap, the Zeno trap (34), positioned between the collision cell and orthogonal acceleration (oa)-TOF section. Through fragment ion trapping, the Zeno trap synchronizes ion release and TOF accelerator pulses. This elevated duty cycle to over 90% and enhanced sensitivity by 4 to 20 fold compared to Q-TOF instruments. diaPASEF, an extension of the PASEF method (35, 36, 37), was developed on Bruker timsTOF instruments and has multiple variations which are elaborated in the following sections.

### MS1-Enhanced Wide-Window DIA

In addition to refining MS2 window designs and leveraging instrument-specific characteristics, certain acquisition schemes aim to enhance MS1 scans for overall data quality improvement, either by m/z range segmentation or inserting MS1 scans per cycle (Fig. 2*D*). Typically applied to Orbitrap instruments, MS1-enhanced DIA leverages high-resolution/accurate mass (HR/AM) measurement with a high dynamic range to foster identification and MS1-based quantification. Examples include WiSIM-DIA (wide selected-ion monitoring DIA) (38, 39), HRMS1-DIA (high-resolution MS1-based quantitative DIA) (40), and BoxCar DIA (41).

WiSIM-DIA, introduced to an Orbitrap Fusion Tribrid instrument in 2014 (38, 39) by the Thermo team, involves parallelly running the Orbitrap for HR/AM MS1 scans at 240 k resolution and the linear ion trap for rapid MS2 scans. Three MS1 scans each spanning 200 m/z range were inserted before the first, 18th, and 35th of 51 MS2 scans per cycle to cover the 400 to 1000 m/z range, resulting in an approximate 3.6 s cycle time. In HRMS1-DIA, a fixed 15 m/z isolation window steps from 400 to 1200 m/z. Among the 54 MS2 scans per cycle, three full m/z range MS1 scans are evenly interspersed, leading to an approximate 5.23 s cycle time and a 1.74 s interval for the chromatogram extraction at the MS1 level.

Within a BoxCar DIA cycle, designed by Sinitcyn *et al.* in 2021 (41), the first phase comprises four MS1 scans, followed by 24 variable windows for MS2 acquisition. The initial MS1 scan is a full scan, with the next three covering one-third of the entire m/z range each. Analogous to the original BoxCar scan proposed by Meier *et al.* (42), the three subsequent MS1 scans in BoxCar DIA also feature non-sequential splitting, partitioning the full m/z range into sequential segments with each MS1 scan focusing on one-third of the segments. This facilitates balanced precursor density at the MS1 level.

### Narrow-Window DIA

All wide-window DIA methods have more or less confronted the challenge of insufficient precursor selectivity or ambiguous precursor-fragment relation arising from the simultaneous fragmentation of precursors within relatively wide windows. Over the past decade, a panel of DIA methods have been developed to address this challenge by reducing the isolation window size while still performing unbiased sampling. A straightforward solution is to employ narrower DDA-like isolation windows of 2 m/z or broader ranges of 2 to 5 m/z.

DIA methods using narrow isolation windows can be further categorized into multi-injection and single-injection types (Fig. 2*E*). Representative methods within the multi-injection category include PAcIFIC (precursor acquisition independent from ion count) (43, 44), PulseDIA (45) and PASS-DIA (46). The latter category encompasses pSMART (47), RTwinDIA (48), and narrow-window DIA approaches (49).

In 2009, Panchaud *et al.* introduced PAcIFIC (43), initially implemented on LTQ Orbitrap XL. By stepping a 2.5 m/z isolation window with a 1 m/z overlap in each cycle, PAcIFIC scans a 15 m/z segment per injection and in total covers the 400 to 1400 m/z range with 67 sample injections. Consequently, a single sample took approximately 4.2 days for data acquisition. In 2011, advancements shortened acquisition to ∼2 days on a faster LTQ Orbitrap Velos instrument (44). PASS-DIA is similar to PAcIFIC, using seven injections to span the 350 to 1400 m/z range with a non-overlapping 2 m/z window, acquiring five MS1 scans and 75 MS2 scans per cycle on a Q Exactive HF instrument (46). PulseDIA, proposed by Cai *et al.* in 2021 (45), requires 2 to 5 injections for the 400 to 1200 m/z range segmented by variable windows (a minimum of 5–11 m/z to a maximum of 11–71 m/z). Notably, PulseDIA isolation windows are non-consecutive - the 28 variably divided segments are subdivided into sub-segments equaling the injection number, with each injection picking a corresponding subsegment.

For single-injection narrow-window DIA, maintaining appropriate cycle times while using narrow isolation windows to cover regular m/z ranges poses challenges to most MS instruments. In 2014, Thermo introduced pSMART (47) on the Q Exactive instrument, employing an approximately 26 s cycle time with five MS1 and 110 MS2 scans per cycle. MS2 isolation windows are variable: 5, 10, and 20 m/z windows for 400 to 800, 800 to 1000, and 1000 to 1200 m/z ranges, respectively. The extended cycle time yields one MS2 spectrum per chromatographic peak. For the quantification purpose, five MS1 scans are interleaved every 20 MS2 scans, yielding approximately 5 s precursor sampling intervals.

An alternative single-injection approach involves reducing the m/z range to be covered. Guided by the peptide mass-retention time relationship, Li *et al.* proposed RTwinDIA in 2018 (48) to demarcate three 2D ranges: 400 to 600 m/z during the first half of the LC gradient, 600 to 800 m/z for 50 to 75% of the gradient, and 800 to 1000 in the final quarter. A 5 m/z window was employed to cover the 200 m/z range, yielding 40 MS2 scans per cycle. On Orbitrap Fusion Lumos, this design requires a cycle time of approximately 3.2 s.

Recently, Thermo Fisher Scientific introduced their latest MS instrument, Orbitrap Astral (50, 51). This new instrument enables parallel MS1 and MS2 acquisition by performing HR/AM MS1 analysis in Orbitrap at 240,000 resolution (at 200 m/z), and MS2 analysis at a ∼200 Hz scan speed using the asymmetric track lossless (Astral) analyzer and at 15,000 resolution on par with Orbitrap. With superior resolving power, analysis speed, and sensitivity, Orbitrap Astral can best fulfill the potential of narrow-window DIA (49). With a 2 m/z single-injection narrow-window DIA strategy covering the 380 to 980 m/z range on this instrument, nearly 10,000 protein groups were detected from HEK293 tryptic digests in triplicate injections. Impressively, approximately 95% of protein groups exhibited coefficients of variation (CVs) under 20%, indicating superior reproducibility.

### Overlapping-Window DIA

A major advantage of narrow-window DIA is the substantial enhancement of the precursor selectivity. However, implementing this type of DIA scheme often leads to a long cycle time on most MS instruments. To cover the entire m/z range with a reasonable cycle time and selectivity in a single-injection manner, a new type of DIA scheme has been designed which incorporates overlapping window arrangements and relies on computational demultiplexing regions for data interpretation (52). As a result, MS2 spectra acquired by overlapping-window DIA can be transformed into cleaner and window size-reduced regions. Three overlapping window designs have emerged: MSX (53) (Fig. 2*F*), intra-cycle overlapping window, and inter-cycle overlapping window (Fig. 2*G*).

MSX, proposed by Egertson *et al.* in 2013 (53), first introduced the DIA acquisition and spectra demultiplexing within an inter-cycle overlapping window scheme. Originally, MSX covered the 500 to 900 m/z range using 20 MS2 scans per cycle. By evenly slicing the whole m/z range into 100 segments, each scan randomly contains five discrete 4 m/z sub-windows. This independently varies sub-window arrangements per cycle, with each segment appearing once per cycle in different scan indices, and two spectra from adjacent cycles have no more than one identical sub-window. Post-acquisition, each 20 m/z MS2 scan is demultiplexed into five 4 m/z regions, increasing selectivity by 5-fold and obviating physical narrow windows. MSX has since expanded as sequential ion accumulation from selected m/z ranges followed by a single MS2 scan, no matter whether windows are random or not. In 2021, Salovska *et al.* introduced BoxCarmax (54) on Orbitrap Fusion Lumos, which incorporated MSX sub-window aggregation and BoxCar-like MS1 division (42) to cover the 357 to 1197 m/z range in only four injections, though this method is not amenable to demultiplexing.

The implementation of MSX requires instruments to store isolated ions from sub-windows, like modern Orbitrap instruments with a quadrupole for isolation and an ion-routing multipole (IRM) equipped alongside the C-trap for storage, which enables concurrent isolation, accumulation, and MS2 scanning (55). Two overlapping-window methods extended demultiplexing to a wider spectrum of instrument platforms. μDIA (microDIA) (56), developed by Heaven *et al.* on Bruker Impact II, uses a 9 m/z isolation window with a 3 m/z overlap (equals to a 6 m/z shift between adjacent windows) to cover the 400 to 1115 m/z range, enabling 120 MS2 scans per ∼3.4 s cycle and resulting in 236 and two demultiplexing regions at 3 and 6 m/z, respectively. Staggered DIA, initially introduced by Searle *et al.* in 2018 (52, 57) on the Q Exactive instrument, alternates the entire m/z range between odd-numbered (500–900 m/z) and even-numbered (490–890 m/z) cycles. Its 20 m/z isolation windows bisect between cycles, yielding 41 10 m/z regions with a demultiplexing factor of 2.

The demultiplexing algorithm introduced with MSX (53) can be applied to all three methods and has been implemented in Skyline (58) and msconvert (59). The basic principle involves solving a non-negative least-squares problem, where the intensity of an expected m/z bin in a real spectrum is represented as a linear combination of intensities from that bin contained in the related demultiplexing regions (Fig. 2*H*). Other tools like Encyclopedia (57) also incorporate their own demultiplexing algorithms.

### Scanning Quadrupole-Based DIA

Unlike the conventional stepping quadrupole, Waters introduced a specialized scanning quadrupole technique in 2017 (60). Using this technique, the quadrupole transmission window is continuously scanned over time by linearly ramping the radio frequency and direct current of the quadrupole rods, which enables quadrupole 1 (Q1) to traverse a desired mass range while simultaneously transmitting precursor ions within the isolation window. Consequently, the use of scanning quadrupole generates an additional Q1 dimension.

When implemented on a Q-TOF instrument, scanning quadrupole allows summing TOF spectra across isolation windows corresponding to pre-defined Q1 m/z bins (Fig. 2*I*). For instance, given 200 Q1 bins of 2.5 m/z spanning the 400 to 900 m/z range and a 20 m/z Q1 window, each bin covers TOF spectra within a 22.5 m/z range, with each TOF spectrum contributing 8 to 9 bins. Summing TOF spectra in this way discretizes the Q1 dimension, making it analogous to an ion mobility dimension. Additionally, for each fragment ion, a trace can be constructed along the Q1 dimension resembling a chromatogram profile (Fig. 2*I*). The Q1 profile apex indicates the precursor m/z for that fragment, with the ideal m/z tolerance equal to the bin size. Regions near the profile center encompass more TOF spectra than edges. Scanning quadrupole can also be viewed as a unique intra-cycle overlapping-window configuration with enhanced precursor selectivity.

Based on the scanning quadrupole technique, SONAR (60) and scanning SWATH (61) were designed on Xevo G2-XS and TripleTOF 6600 instruments, respectively. Specifically, in the SONAR method developed by Waters in 2017, MS1 and MS2 acquisitions alternate using the same quadrupole time to scan across the 400 to 900 m/z range. Unfragmented precursor ions are subjected to a low-energy collision energy, while fragment ions experience high-energy ramping. The resulting Q1 dimension consists of 200 bins, each spanning a 2.5 m/z width. In scanning SWATH developed by Messner *et al.* in 2021, MS2 spectra were acquired within the 400 to 900 m/z range and binned with a Q1 size of 2 m/z.

### diaPASEF

Trapped ion mobility spectrometry (TIMS) is an IMS technique that allows for the control of ion motion in the gas phase, enabling axial trapping and releasing of ions in the TIMS tunnel (62). In TIMS, ions with higher m/z values generally exhibit lower mobility (higher 1/*K*_*0*_), leading to their earlier release from the TIMS analyzer compared to ions with lower m/z values.

In 2015, Meier *et al.* introduced the parallel accumulation-serial fragmentation (PASEF) method, an instrument control strategy to step the quadrupole as a function of the TIMS ramp time (35). PASEF substantially elevates the sampling rate of precursor ions injected from the ion source. When implemented on a TIMS-Q-TOF instrument, PASEF achieved nearly 100% acquisition of fragment ion currents from low-complexity samples (35). Later in 2017, Bruker launched timsTOF Pro equipped with a new dual TIMS analyzer, in which two TIMS devices operate in tandem: one accumulates ions, while the other performs TIMS scanning simultaneously (36). The short transfer time between these regions achieves a nearly 100% duty cycle.

**Fig. 3:**
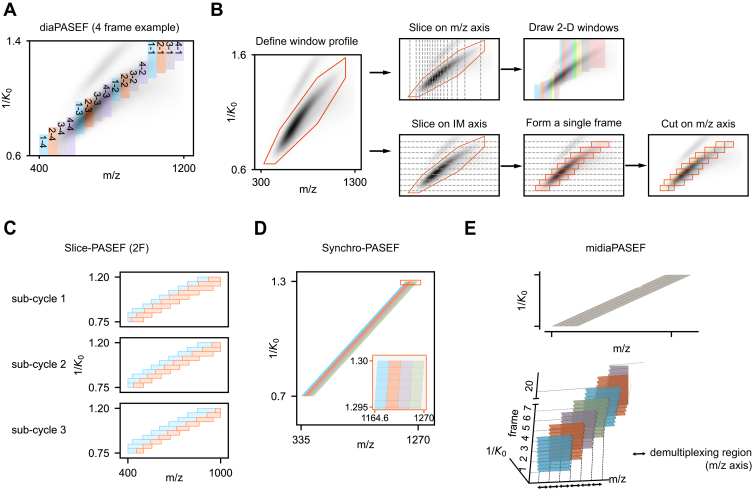
**PASEF-enhanced DIA acquisition schemes.***A*, example of a 4-frame diaPASEF scheme showing 2-D isolation windows distributed across m/z and ion mobility dimensions. *B*, two procedures for forming 2-D windows on the m/z-ion mobility plane. Windows can be sliced first on the m/z axis and then drawn within each segment (*upper panel*) or vice versa after slicing the IM axis (*lower panel*). In the latter case, a single frame is formed, followed by slicing each window on the m/z axis. *C*, example of the 2-frame Slice-PASEF with three subcycles. Each subcycle covers the same m/z and IM ranges but differs in the m/z cutting point(s) in each window. *D*, example of the 4-frame Synchro-PASEF. The IM axis is sliced into 927 segments over the 100 ms TIMS ramp time, with four frames arranged adjacently. *E*, the midiaPASEF scheme has an IM axis sliced into 927 segments and 20 frames defined per cycle. Adjacent frames overlapped by two-third of the m/z range on each IM slice (*upper panel*), and a 3-D view of the overlapping windows from different frames (*lower panel*). The windows with the lowest 1/*K*_*0*_ are illustrated as an example of overlapping with a 3-fold demultiplexing factor. PASEF, parallel accumulation-serial fragmentation.

In 2020, Meier *et al.* introduced diaPASEF to extend PASEF to DIA (33). Specifically, diaPASEF defines 2-D acquisition windows with 1 m/z dimension and another mobility dimension which is related to the desired m/z range for a given window. A full TIMS scan, termed a diaPASEF scan or a frame, indicates one complete release of ions from the TIMS analyzer. Given the distribution of the precursor ion cloud, there are usually more than one 2-D acquisition windows within each frame. A total of six diaPASEF acquisition schemes with varying window arrangements were used in the original work by Meier *et al.* For instance, the high sensitivity scheme involved four diaPASEF scans per cycle, each with four steps, yielding a total of 16 windows arranged in one row (Fig. 3*A*). The standard scheme featured 16 diaPASEF scans per cycle, each with four steps, totaling 64 windows arranged in two rows. Using the 16-diaPASEF scan scheme, Meier *et al.* identified 66,998 peptides associated with 7800 proteins in triplicate injections from 200 ng HeLa tryptic digests. In addition, the use of TIMS for DIA has been shown by Charkow and Röst to achieve an equivalent 4-fold reduction in the size of the Q1 isolation window (from 25 m/z to 6.25 m/z) (63).

Building upon the principles of diaPASEF, various window arrangements have been devised to cater to different analytical needs by phosphoproteome analysis (64) or short-gradient MS analysis (65). A general approach for designing diaPASEF windows can be outlined in three key steps (Fig. 3*B*): framing the desired acquisition area according to the precursor ion cloud; slicing on the m/z axis; and defining 2-D windows in each m/z segment. This procedure usually results in acquisition windows more finely sliced in the m/z dimension while coarsely sliced in the ion mobility (IM) dimension. In 2022, Skowronek *et al.* released a Python package py_diAID to assist in the optimization of 2-D acquisition windows (66), which achieved a 6% increase in identified peptides from regular proteomic samples and a significant gain of phosphosites (28%) from phosphoproteomic samples.

### Recent Development of PASEF-Enhanced DIA

Several recently developed PASEF-enhanced DIA methods, including Synchro-PASEF (67), Slice-PASEF (68), and midiaPASEF (69), have implemented a fine segmentation of the IM dimension and an m/z overlap between acquisition windows and frames. Thus, one precursor ion can be included in more than one window with varying m/z ranges. The window design of these methods all involves four steps (Fig. 3*B*). First, a single-frame acquisition area is defined as a quadrilateral or polygon profile that covers the expected precursor ion cloud. Then, the IM axis is (typically evenly) sliced into *n* segments to form a preliminary single frame containing *n* 2-D acquisition windows. Further, for each IM segment, the covered m/z range is divided into *k* pieces. Finally, by picking either 1 m/z piece or several contiguous m/z pieces from each IM segment, *h* frames are generated.

Slice-PASEF, introduced by Szyrwiel *et al.* in 2022 (68), defines three acquisition schemes: 1-frame (1F), 2F, and 4F. In the 2F scheme, 10 IM segments are sliced and each segment is further divided and allocated to two frames (Fig. 3*C*). Notably, for each initial window, the covered IM range and m/z range remain fixed. However, the m/z cutting point is shifted across three cycles: the second cycle has cutting points right-shifted relative to the first, and the third cycle has points further right-shifted. This process of “expanding and shrinking” the m/z range results in dynamic window arrangements with a pre-defined scheme. The Slice-PASEF concept can be extended to a family of multi-frame (MF) schemes. Any MF scheme with a minimum of two frames naturally incorporates inter-cycle overlapping windows, which may facilitate subsequent data analysis.

Both Synchro-PASEF (67) and midiaPASEF (69) utilize 927 IM segments when employing a 100 ms TIMS ramp time. Each IM slice closely corresponds to an approximate 110 μs TOF pulse period. This synchronization enables the quadrupole position to continually traverse the m/z range, coordinating with TOF pulses and their associated m/z ranges. Synchro-PASEF, developed by Skowronek *et al.* in 2022, introduces three schemes with variations in the frame count and isolation window. For instance, the first scheme comprises four frames, each with a fixed 25 m/z window width, resulting in 100 m/z coverage per IM segment. Adjacent windows within a frame have a 0.9 m/z shift, leading to mainly intra-frame overlapping windows. This configuration covers ∼935 m/z in one cycle (Fig. 3*D*). On the other side, midiaPASEF, proposed by Distler *et al.* in 2022, incorporates more evident inter-frame overlapping windows. Here, a fixed 36 m/z window is used across all frames, while adjacent windows in an IM segment shift by 12 m/z (Fig. 3*E*). This introduces a demultiplexing factor of three in the m/z dimension alone, achieving the most fine-grained signal segmentation among current methods, as indicated by its name "maximizing information content in DIA-PASEF".

All three methods, namely Slice-PASEF, Synchro-PASEF, and midiaPASEF, enable precursor ions to be selected in varied windows. Thus, the inter-connected fragment ion signals obtained across different windows offer additional information for detecting real signals while mitigating potential chemical and electrical noises.

### Unconventional Methods

In this category of acquisition schemes adopting irregular cycles, mixed-mode DIA methods incorporate additional schemes like DDA and targeted acquisition alongside DIA. Examples include MMA (70), DDIA (71), and Hybrid-DIA (72).

MMA (multi-mode acquisition), introduced by Waters in 2016 (70), represents an unconventional acquisition method implementing multiple DIA schemes including wide-window, narrow-window, and overlapping-window. Through dynamic adjustments by the acquisition scheduler system, MMA also incorporates DDA and targeted acquisition. MMA aims to sample as many precursors as possible while limiting their co-fragmentation, which is expected to reduce MS2 spectral complexity and facilitate computational demultiplexing.

In 2020, Guan *et al.* introduced DDIA (71), which inserts DDA MS2 scans between MS1 and DIA MS2 scans per cycle. Splitting spectra enables construction of a small run-specific library with accurate RTs from DDA data. This library can refine fragmentation and RT prediction to assist in proteome profiling based on DIA data.

Hybrid-DIA, introduced by Martínez-Val *et al.* in 2023 (72), implements a two-step targeted acquisition prior to wide-window DIA scans. In each cycle, an MS1 spectrum is first acquired and analyzed to detect heavy-labeled peptides. If expected signals are present, parallel reaction monitoring (PRM) scans are triggered, followed by MSX scans targeting pre-defined light-labeled peptides. Compared to wide-window DIA alone, the two-step triggered MSX scan provides an ∼8-fold increase of signal-to-noise ratio for the top three fragment ions. In a benchmark dataset, hybrid-DIA enabled a two-fold increase of quantifiable targeted phosphopeptides.

While most DIA methods are applied to LC-MS/MS platforms, DI-SPA, introduced by Meyer *et al.* in 2020 (73), achieved DIA data acquisition for direct-infusion MS. In this method, gas-phase separation of precursor ions occurs in both FAIMS and quadrupole. The use of FAIMS in conjunction with narrow window acquisition reduces MS2 spectra complexity and the application of stepping compensation voltages further increases the precursor sampling sensitivity. As a result, DI-SPA allowed superfast identification and quantification of nearly 500 targeted proteins within minutes (∼3.5 proteins per second) across 132 samples.

## Strategies for DIA Data Analysis

**Fig. 4:**
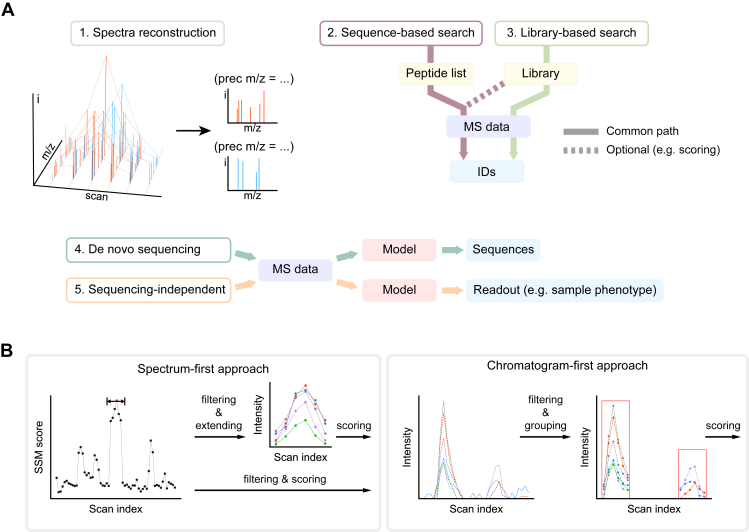
**Strategies for DIA data analysis.***A*, five categories of data analysis strategies as defined in this review. *B*, specification of sequence-based and library-based searches with two approaches. In the spectrum-first approach, spectrum-spectrum match (SSM) scores are calculated along the chromatogram to find the best spectrum matching for precursors. Peak groups are formed from the seed spectrum and its adjacent spectra. In the chromatogram-first approach, extracted ion chromatograms are built prior to peak grouping if enough local traces are concentrated in one region.

The substantial complexity of DIA spectra resulting from the co-fragmentation of precursor ions has posed a significant challenge to spectral deconvolution or peptide sequence identification. A wide array of software tools has been developed to tackle this challenge (Table 1), which implement one or several major strategies as we define below: spectrum reconstruction, sequence-based search, library-based search, *de novo* sequencing, and sequencing-independent (Fig. 4*A*). Furthermore, the library-based search can be performed in either a spectrum-first or chromatogram-first manner, while the sequence-based search is conducted in a spectrum-first manner (Table 1). The following sections introduce these strategies designed for DIA data analysis and overview different software tools developed based on specific strategies.

**Table 1:** DIA data analysis tools

Software	Strategies			x-first approach	
	Spectra reconstruction	Sequence-based	Library-based	Spectrum	Chromatogram
Ion accounting (96)	✓	✓^a^		✓^b^	
ETISEQ (98)	✓				
DeMux (74)	✓				
DIA-Umpire (75)	✓		✓		✓^b^
Group-DIA (99)	✓		✓		✓^b^
Dear-DIA^XMBD^ (100)	✓				
FT-ARM (28)		✓	✓	✓	
MSPLIT-DIA (93)			✓	✓	
PECAN (Walnut)		✓		✓	
Specter (102)			✓	✓	
EncyclopeDIA (57)			✓	✓	
FIGS (103)			✓	✓	
CsoDIAq (73)			✓	✓	
DIAmeter (81)		✓		✓	
MSFragger-DIA (82)		✓		✓	
Skyline (58)			✓		✓
OpenSWATH (83)			✓		✓
DIANA (111)			✓		✓
SWATHProphet (119)			✓		✓
DIA-NN (84)			✓		✓
MaxDIA (41)			✓		✓
DreamDIA^XMBD^ (123)			✓		✓

(1) All software tools in this table except ion accounting are open-access. Commercial ones are not included here; (2) DeepNovo-DIA (89), which implements the strategy of *de novo* sequencing, and DIA tensor (91) and mstc (92), which implements the sequencing-independent strategy, are not listed here.

a The sequence-based search strategy implemented in ion accounting aims to handle reconstructed spectra instead of raw DIA spectra.

b Three tools that implement spectra reconstruction can also perform sequence- or library-based database search with a specific x-first approach.

### Spectra Reconstruction

Spectra reconstruction aims to deconvolute raw DIA MS data through clustering potentially related fragment ion signals or pairing precursor-fragment ion signals. These signals are then extracted to generate new deconvoluted MS2 spectra, which may or may not contain precursor m/z information. This spectra reconstruction procedure is often referred to as the generation of DDA-like pseudo spectra from demultiplexed DIA data, which is employed by software tools like DeMux (74) and DIA-Umpire (75, 76). One advantage of performing spectra reconstruction is the seamless application of methods well-established for DDA data analysis such as open search or *de novo* sequencing to DIA data analysis when the precursor m/z information is available.

### Sequence-Based Search

Sequence-based search is predominantly used for processing DDA data in several classical search engines like SEQUEST (77), as well as modern ones like pFind (78) and MSFragger (79). This strategy, also widely applied to DIA data analysis in the early days (19, 26), has recently regained attention. Software tools such as PECAN (80), DIAmeter (81), and MSFragger-DIA (82) have implemented the sequence-based search, also referred to as the direct search. One distinct feature of sequence-based search is to directly search undeconvoluted DIA spectra against a peptide sequence database, without heavily relying on prior information or MS2 spectra pre-processing. Notably, sequence-based search can also leverage the peptide fragmentation pattern and retention time (RT), or a targeted peptide list in the analysis workflow, to enhance the identification accuracy and sensitivity (Fig. 4*A*).

### Library-Based Search

Library-based search represents the most widely employed strategy that incorporates prior information at the outset of DIA data analysis. Typically, the data search is restricted to a defined precursor ion list in a library. The prior information may encompass various parameters, including but not limited to peptide fragmentation patterns, peptide RT values, precursor ion mobility values, and peptide MS detectability (*e.g.*, ionization efficiency and ion transmission efficiency). The majority of currently used DIA analysis tools fall under this category, such as OpenSWATH (83), Skyline (58) and DIA-NN (84).

### *De Novo* Sequencing

*De novo* sequencing is an intriguing technique in MS data analysis, with special values for tasks like immunopeptide identification, and it has been extensively explored in DDA data analysis (85, 86, 87, 88). However, applying *de novo* sequencing to DIA data analysis presents a unique challenge due to the absence of explicit mass restrictions when generating peptide sequences from MS2 spectra. In principle, *de novo* sequencing can be implemented on DIA data processing when the spectra have been deconvoluted using a spectra reconstruction approach, or when the DIA data is acquired using a DDA-like narrow window. Alternatively, DeepNovo-DIA (89), a tool dedicated to *de novo* sequencing for DIA data, relies on feature detection and the determination of potential precursor-fragment ion pairs without spectra reconstruction.

### Sequencing-Independent

All the above data analysis tools report either deconvoluted spectra or peptide/protein sequencing results. However, there exists another approach to utilize raw MS data to extract useful knowledge while skipping the sequencing step. In 2012, Palmblad and Deelder introduced compareMS^2^ (90), which builds a phylogenetic tree by calculating spectral similarity as a distance matrix from DDA raw data acquired from samples of various species. This approach has the potential to avoid bias arising from sequence annotation. In 2020 and 2021, Zhang *et al.* and Cadow *et al.* extended this sequencing-independent concept to DIA data analysis by developing DIA tensor (91) and mstc (92), respectively. Both tools aim to build end-to-end models with MS raw data as input and to predict sample phenotypes such as the disease state as output.

### Spectrum-First *Versus* Chromatogram-First in Library-Based Search

Depending on which step to use the chromatogram information in the entire data analysis pipeline, the library-based DIA data search can be specified to operate in a spectrum-first or chromatogram-first manner.

In the spectrum-first manner, spectrum-spectrum matching (SSM) between an experimental MS2 spectrum and a library spectrum, as described by Wang *et al.* (93), is the fundamental unit of data analysis. The library fragments (or theoretical fragments) of one peptide precursor are usually treated as a whole to possibly serve as a vector during spectra matching. Then an SSM score-based chromatogram is yielded for each queried precursor ion, indicating the quality of SSMs (Fig. 4*B*). Conversely, data search in the chromatogram-first manner treats fragment ions as individual units and usually constructs their extracted ion currents (XICs) separately at the first step (Fig. 4*B*). Because extracting the chromatogram information is pivotal in DIA data analysis, most tools operating in the spectrum-first manner also reconstruct fragment XICs from a seed within an SSM trace by integrating adjacent scans after the initial spectrum matching and filtering, which can be also referred to as chromatogram-delayed.

The spectrum-first and chromatogram-first approaches employed in library-based searches mainly differ in two aspects. First, the spectrum-first approach usually implements pre-filtering on SSMs and selects the best SSM for fragment XIC construction, while the chromatogram-first approach tends to find the best combinations of peak traces to form fragment peak groups for each quired precursor ion. Second, the single-spectrum matching quality is more important for the spectrum-first approach, while it only acts as a component to be aggregated in one or more sub-scores for the chromatogram-first approach. The library-based search can be performed with either approach, whereas the sequence-based search generally relies on the spectrum-first approach. This may be attributed to the hardware challenge in building XICs for all possible fragment ions without the guidance from either the restricted number of fragment ions or the possible retention time range for signal extraction. Furthermore, in the context of LC-IMS-MS/MS data, an analogous trace-first approach can be defined to replace the chromatogram-first approach.

It is noteworthy that the two peak grouping approaches defined in our review are complementary to the widely used peptide-centric analysis (94) in specifying workflows implemented in various tools. For instance, OpenSWATH (83), originally classified as peptide-centric, can be also viewed as chromatogram-first, given that OpenSWATH constructs potential peak groups using high-intensity peaks derived from the initial fragment XIC building step. These groups are scored with each peak group as the basic scoring unit, followed by the training of an XGBoost model for discrimination in target-decoy competition. As for PECAN (80) which is also recognized as peptide-centric, it is classified to be spectrum-first here considering its use of a modified dot product as a preliminary score. Subsequently, rule-based filtering and auxiliary score calculation for each SSM are applied in PECAN to facilitate target-decoy competition by Percolator (95). The spectrum- or chromatogram-first approach can be further combined with library- or sequence-based analysis strategies for a precise description of an analysis workflow, as illustrated in the following section.

## Software Tools for DIA Data Analysis

Based on the incorporation and combination of major data analysis strategies as defined above, herein we review specific designs and key features of data analysis tools that can be classified into four classes: spectra reconstruction, library-based and spectrum-first, library-based and chromatogram-first, and sequence-based and spectrum-first.

### Spectra Reconstruction

Spectra reconstruction-based data analysis comprises three major steps: pre-processing and feature detection, ion peak grouping, and spectra generation. First, discrete signals within a defined m/z tolerance are traced along the chromatogram axis, with or without filtering out potential noise. Ion peak grouping is then implemented to relate precursor and fragment ion signals or correlate two fragment ion signals based on their ion trace similarities. Once these signal relationships are determined, the raw signals are extracted and aggregated to form deconvoluted spectra, with optional adjustments to enhance peptide identification (Table 2).

**Table 2:** Major characteristics of spectra reconstruction tools

Software	Preprocessing	Ion grouping criteria	Fragment assignment	Other functions
Ion accounting (96)	Ion detection based on 2D-convolution	Apex RT	One-to-multi (reassigned in sequence-based search step)	Sequence-based search
ETISEQ (98)	Potential contaminant signal removing	PCC and cross-correlation lag	(1) One-to-multi; (2) unmatched fragments are assigned to all precursors	/
DeMux (74)	Coarse spectral binning	(No precursor signal required) correlation between m/z bins in MS2 spectral map	/	/
DIA-Umpire (75)	Interpolation and unimodal peak splitting	(1) summed mass of two fragments equals to precursor mass; or (2) precursor-fragment PCC rank and apex RT	One-to-multi	Library-based search
Group-DIA (99)	Cross-run spectra alignment	k-means-based precursor-fragment clustering	One-to-one	Library-based search
Dear-DIA^XMBD^ (100)	Fragment XIC representation encoding	k-means-based fragment clustering with CNN calculated XIC similarity	One-to-one	/

The Ion Accounting algorithm (96), developed for MS^E^ data analysis, first detects potential ions by applying a 2D convolutional filter on a mass spectral map. Local maxima exceeding a defined threshold indicate the presence of an ion (97). It then groups ions by identifying fragment ions with apex retention time (RT) deviations below a threshold for each precursor ion (18). This generates a table of associated precursor-fragment ions, annotated by properties like monoisotopic mass, aggregated peak area, and apex RT. A sequence-based search is performed based on this table to allow peptide identification in 3 passes. Fragment signals correlated to multiple precursors are initially assigned to all, then reassigned according to the match quality (96).

ETISEQ (98), designed for full-scan DIA data analysis, first removes possible contaminant signals. After constructing XICs from the filtered data, it associates fragments with precursors based on their correlation in the chromatogram axis. For spectral generation, ETISEQ extracts signals from the spectrum of apex RT. Fragments matched to multiple precursors are assigned to multiple spectra, while unmatched fragments are assigned to all spectra within the RT range.

DeMux (74) works on the mass spectral map with coarse spectral binning (1.0005 m/z in the original paper). Within each concatenated chromatogram block containing spectra from a common isolation window, it groups ions without initial feature detection. Since MS1 data processing is avoided, DeMux uses the most intense bins as seeds to identify other highly correlated bins. It then generates 1D convolutional filters to reduce noise and penalize co-fragmentation signals. For spectral generation, DeMux extracts signals from the raw data based on the pre-determined m/z and RT ranges. It then aggregates the final signal intensities across adjacent scans using the convolutional filter.

As a comprehensive spectra reconstruction-based analysis framework, DIA-Umpire (75, 76) enables generation of pseudo MS2 spectra amenable to DDA data search, followed by DIA data search based on the prior identification output. For feature detection, signals within a defined m/z tolerance are first traced and interpolated before the traces are split into unimodal peak curves. For ion grouping, precursor ions serve as seeds and are categorized into different quality tiers according to their isotopic signals in MS1 or MS2 spectra. Fragment assignment for each precursor mainly relies on the sum of fragment masses, or chromatogram similarity ranking and apex RT deviation. Furthermore, DIA-Umpire can modify the pseudo spectra by adding complementary ions or adjusting ion intensities for enhanced identification.

For proteomic experiments analyzing samples of similar composition with a specific acquisition method, Group-DIA (99) leverages signals commonly detected in multiple runs by first aligning them by dynamic programming, using spectral similarity as the metric. After alignment, a reference run guides the XIC extraction for other runs. Meanwhile, ion peak correlations are performed experiment-wide by concatenating XICs for the same ions across runs. Fragment ions are clustered into different groups to determine precursor-fragment relations. Group-DIA also allows for library-based data search by assigning specific prior probabilities to targeted peptides.

To address the noise and XIC misalignment issues, Dear-DIA^XMBD^ (100) utilizes a variational autoencoder model to extract latent representations of fragment XICs, and these latent features in a consistent space are then k-means clustered based on Euclidean distance. Meanwhile, a precursor list with possible fragments generated from a protein sequence database are used to guide the pairing of fragment clusters with candidate precursors. To form a new deconvoluted spectrum, each selected fragment group relies on an additional similarity score calculated by a convolutional neural network.

### Library-Based & Spectrum-First

This class of software tools operating in a spectrum-first manner prioritizes the spectrum-spectrum match (SSM) in library-based searches (Table 3).

**Table 3:** Major characteristics of tools performing library-based searches in a spectrum-first manner

Software	Primary SSM score	Used library information	
		Fragmentation pattern	RT
FT-ARM (28)	Dot product	Optional	
MSPLIT-DIA (93)	Cosine similarity	✓	Optional
CsoDIAq (73)	Cosine similarity	✓	
Encyclopedia (57)	Product of summed correlation-weighted dot products and the factorial of the number of matched ions	✓	✓
Specter (102)	Coefficients of linear combination of library spectra	✓	✓
FIGS (103)	Coefficients of linear combination of library spectra	✓	✓

FT-ARM (28) generates theoretical fragments from a peptide sequence database or directly uses a spectral library. It calculates dot products of compared spectra as SSM scores to trace match quality along the chromatogram axis. The spectrum with the highest SSM score serves as potential evidence for precursor ion detection. Notably, when library fragments have a fixed intensity of 1, SSM traces become quantitative with under-curve areas reflecting the aggregated fragment intensities. SSM scores also enable target-decoy-based error estimation.

MSPLIT-DIA (93) first matches and scores the library spectra and experimental DIA spectra using cosine similarity. To exclude queried precursors sharing too many fragments, only the SSM of the top cosine similarity is retained. These spectra then support XIC building for top fragments by extending adjacent spectra for each precursor. The final precursor score is the product of maximum SSM cosine similarity and mean fragment-fragment ion similarity. Later on, Cranney and Meyer introduced CsoDIAq (101) similar to MSPLIT-DIA to enable quantification without XIC reconstruction in the analysis of DI-SPA data (73).

Encyclopedia (57) performs a library-based search using SSM scores defined as the product of correlation-weighted dot products and the factorial of the number of matched ions. After determining the best SSM per precursor ion, it calculates auxiliary scores for FDR estimation, and adjusts certain scores based on the library fragment ion frequency. Encyclopedia implements a two-pass search strategy: the first pass only uses spectral information from the library, while the second pass incorporates RT scores. Before the second pass, 2-D kernel density estimate-based library RT alignment is implemented together with a mixture model for outlier detection.

For a high-quality library with spectra precisely matching the DIA data, Specter (102) represents each MS2 spectrum as a linear combination of library spectra. SSM scores are coefficients solved *via* non-negative least squares. All precursor SSM traces are filtered to find local traces containing at least five consecutive SSMs and a local maximum. The maximum scoring SSM is retained to calculate sub-scores and discriminative scores that are used for error rate estimation. Peak quantification is derived from the smoothed SSM score trace. FIGS (103), similar to Specter, first identifies precursors with enough unique fragment ions while precursors lacking unique fragments undergo further iterative searches. A key feature of both tools is their ability to deconvolute DIA MS2 spectra even from concurrently fragmented isomeric peptides by leveraging a high-quality library.

### Library-Based & Chromatogram-First

This class constituting the largest group of currently used tools for DIA data analysis share a 4-step workflow by firstly inputting a library and raw DIA data. The library is typically pre-processed to remove unexpected fragments and add decoys. Next, the library is fitted to the experimental DIA data in a preliminary search to calibrate mass errors and align RTs. A main search is then performed to construct XICs for all library precursor and fragment ions. The XICs are split into candidate ion peaks, which are aggregated into putative peak groups for scoring, error control, and quantification (Table 4).

**Table 4:** Major characteristics of tools performing library-based searches in a chromatogram-first manner

Software	Basic scoring unit	Scoring model
Skyline (58)	Peak group	(mProphet) linear classifier; (Avant-Garde) combination of subscores with fixed parameters
OpenSWATH (83)	Peak group	(PyProphet) XGBoost
DIANA (111)	Peak group	(PyProphet) XGBoost
SWATHProphet (119)	Peak group	mProphet-like linear classifier
DIA-NN (84)	One best peak	Linear classifier and fully connected neural network
MaxDIA (41)	Peak group	XGBoost
DreamDIA^XMBD^ (123)	Raw XICs with a fixed length	LSTM neural network and XGBoost

Skyline, initially developed for SRM data analysis, has been adapted to DIA data analysis (58, 104, 105). Owing to the continuous efforts of the developer team, Skyline supports nearly all data types acquired on different instruments and by various acquisition methods. For final discriminative score assignment, either a combination of pre-defined fixed coefficients or mProphet-based semi-supervised linear discriminant analysis can be implemented (106). The Avant-Garde plug-in enables additional score calculations and aggregation for error control and peak boundary adjustment (107). Supporting diverse plug-ins (108) and integrating third-party tools, together with its user-friendly graphical interface, have made Skyline a versatile and comprehensive platform offering a full-fledged DIA data analysis solution.

Originally developed for SWATH, OpenSWATH (83) built on the OpenMS platform (109, 110) now also supports data analysis for SONAR, diaPASEF, and other DIA schemes. Its integrated workflow enables automatic signal extraction, RT calibration, and scoring of queried peptides. Downstream statistical error control initially relied on mProphet (106), and later switched to the updated PyProphet (111, 112) which incorporates *a priori* probability π_0_ to reflect the fraction of undetectable library entries (113, 114), and estimates error rates on multi-levels. The functionality of OpenSWATH has been further diversified by incorporating tools like IPF for PTM scoring (115), TRIC (116) and DIAlignR (117) for multi-run alignment, Mobi-DIK for diaPASEF support (33), and GproDIA for glycoproteomics (118). DIANA (111), similar to OpenSWATH, features a unique Markov ratio probability score for the “soft” measure of spectral similarity between precursor isotopes or fragment pairs based on intensity ratios across two XICs. SWATHProphet (119) also works in a standard workflow and is a part of the Trans-Proteomic Pipeline (TPP) (120, 121), allowing seamless integration with other TPP tools like ProteinProphet (122).

DIA-NN follows the standard workflow with certain unique designs (84). One specific feature is its minimal scoring unit, the best peak, which is selected per peak group based on summed correlations with other peaks. Most scores are calculated based on this best peak to avoid introducing bias from low-quality peaks. Another feature is the two-step scoring for peak groups and queried library entries. First, pairwise target-decoys are used to calculate the sub-score differences to establish a linear regression model to assess the peak group quality. In the second step, peak group sub-scores are propagated to precursors, with final detection scores learned by a fully-connected neural network with cross-entropy loss function. The training of network only requires one epoch, which avoids potential over-fitting. Notably, in both steps, DIA-NN utilizes all data points from targets and decoys, unlike most current tools such as Percolator (95) and PyProphet (112) leveraging a portion of data in each training iteration.

Unlike typical library-based search workflows, MaxDIA (41) achieves feature detection and deisotoping for both MS1 and MS2 spectra from DIA data. This offers the advantage of eliminating potential signal reuse across peak groups, especially for peptides sharing similar sequences yet varying on residues or PTM localization sites. Moreover, MaxDIA implements “bootstrap DIA” with six total “first search” stages to calibrate library RT, IM, and raw data mass by both linear and non-linear alignments. After calibration, the processed data and library are matched and scored to train a target-decoy discriminative model.

As artificial metrics used by most analysis tools may insufficiently represent available information in DIA data, DreamDIA^XMBD^ (123) uses XICs directly as neural network inputs to learn scores. For each queried precursor, it extracts a total of 170 XICs based on available fragments and precursor/fragment isotopes from an experimental library or theoretical predictions. The network outputs are then used for RT alignment and peak-picking, or concatenated with other sub-scores to train an XGBoost-based discriminative model.

### Sequence-Based & Spectrum-First

In the sequence-based search, the lack of guidance from fragment intensities in a library makes SSM scores more vulnerable to fragments with shared m/z values. In view of this challenge, PECAN (80) exploits both a targeted and background peptide lists to evaluate the contribution and randomness of fragment matching (Table 5). First, the intensities of theoretical fragments generated from both background and targeted peptide lists are divided by the counts of similar m/z ions. This procedure reduces the influence of frequently occurring fragments on SSM scoring. Second, decoys are generated from the background peptide list to determine background scores per isolation window by estimating the quality of random matches. For each targeted precursor ion, after deducting the window background score for the SSM dot product trace, the ultimate SSM score is calculated as the mean of calibrated scores from a local trace. Identified peptides are then filtered by the ratios of high-quality matched fragment ions and FDR is estimated by Percolator.

**Table 5:** Major characteristics of tools performing sequence-based searches in a spectrum-first manner

Software	Primary SSM score	Matching restriction
PECAN (80)	Background score-subtracted dot product	Reduce fragment matching importance based on frequency
MSFragger-DIA (82)	MSFragger hyperscore (79)	(1) Maximum PSMs per spectrum; (2) High PSM score-first signal picking
DIAmeter (81)	XCorr	(1) Maximum PSMs per charge state per spectrum; (2) PSM elimination based on combined subscores compared to the PSM with the highest primary score

MSFragger-DIA (82) and DIAmeter (81), two tools that are built upon the DDA search engine MSFragger (79) and Tide (124), conduct direct search of DIA spectra against fragments generated from a given peptide database. MSFragger-DIA first performs deisotoping and a preliminary search for mass calibration. It then executes a full search to acquire SSMs without using chromatogram information. For each spectrum, the maximum number of candidate matches is restricted to user-defined values, differing for wide-window or narrow-window DIA data. XICs are subsequently constructed to filter out qualitatively invalid ions. Using median apex RTs as baselines, MSFragger-DIA removes certain fragment ions with abnormal RTs, or eliminates PSMs with outlying precursor ions. To handle shared fragment ions among different precursors, a greedy strategy associates all possible fragment ions to each precursor. Residual spectra with matched signals removed are then utilized iteratively for the next precursor, in a PSM hyper-score-sorted order. Finally, matching results can be processed with the FragPipe (125) workflow comprising MSBooster (126), Percolator/PeptideProphet (95, 127), ProteinProphet (122), Philosopher for report filtering (128), and EasyPQP for library generation.

DIAmeter (81) also restricts the maximum number of candidate matches per spectrum, defaulting to five precursors for each charge state from 1 to 5, ranked by the primary XCorr score (129). After matching and XIC construction, sub-scores are calculated for the matches. PSMs are preliminarily filtered based on the aggregated score derived from the top-scoring match per spectrum and charge state. Percolator is then used to train a discriminative model without limiting to one PSM per spectrum.

Apart from the outlined tools specifically designed for DIA data analysis, other software packages initially developed for processing chimera spectra (130) in DDA data, have the potential in handling multiplexed DIA spectra. One such example is ProbIDtree (131) working in a sequence-based searching mode. Additionally, Open-pFind (132), owing to its ability in both processing chimera spectra and performing an open search, has demonstrated the feasibility in narrow-window DIA data analysis (48). We anticipate functional expansion of Open-pFind could facilitate DIA data analysis with dedicated workflows.

### Commercial Software

Commercial software usually offers user-friendly graphical interfaces, streamlined analysis workflows, and auxiliary functions for spectral inspection and downstream analysis. In addition to the vendor-developed software tools such as PLGS (96) by Waters, PeakView (29) by SCIEX, and ProteoScape by Bruker, those provided by companies specialized in informatic services such as Spectronaut (30) by Biognosys, PEAKS (133) by Bioinformatics Solutions, and Scaffold DIA (134) by Proteome Software, also have gained popularity in the DIA proteomics field.

Of note, these commercial products often undergo frequent updates to boost their performance continuously. For instance, Spectronaut has become a widely used DIA analysis package, introducing the directDIA workflow from version 11, PTM localization scoring from version 13 (135), and providing advanced machine learning support for many data analysis steps in recent updates. Since 2018, a DIA sequence searching module was incorporated to PEAKS from version X and a library searching module was added to version Xplus. Moreover, it has a unique feature to conduct DIA *de novo* sequencing, supported by their DeepNovo-DIA development (89).

## Working with Libraries in DIA Data Analysis

Generally, a typical library contains two main types of information: a precursor ion list and the associated precursor ion attributes. The former defines a maximum search space, restricting feature detection and peak group scoring (136, 137, 138), while the latter including the fragmentation pattern, peptide RT, and ion mobility is generally required for signal extraction and spectral matching assessment.

Fragmentation patterns serve dual roles, restricting the fragments to be used in signal extraction, and providing relative intensities for scoring (139, 140, 141). Fragmentation patterns are relatively stable under specific instrument settings (142) but can vary across platforms and fragmentation modes (143, 144). Precise RT values also restrict signal extraction within the library-defined RT ranges, although they are more variable than fragmentation patterns due to instrument fluctuations. To enable RT alignment across runs, indexed RT (iRT) values are usually determined using spiked-in synthetic standards like Biognosys iRT peptides (145) and PROCAL peptides (146), or common endogenous peptides like CiRT peptides (147). RT calibration can also be achieved on high-scoring peptides from a preliminary search, independent of iRT peptides (41, 84). Compared to RT, IM exhibits a much higher stability in a given mobility analyzer such as TIMS, and is usually transferable between experiments (148).

In recent years, diverse approaches have been established for library generation. Broadly, libraries can be derived from experimental MS data, computational prediction, or through a combination. Common approaches acquire additional experimental data from the samples to be analyzed, including offline fractionation combined with DDA analysis to yield deep coverages at the expense of large sample consumption, or narrow-window DIA offering a more economical alternative with less deep sampling (149). In addition, such fractionation-derived DDA data and narrow-window DIA data are well-suited for fine-tuning models to predict library information. Meanwhile, the accumulation of data from numerous MS-based proteomics studies have provided a vast shared resource, and many large-scale libraries generated for model organisms (24, 150, 151, 152) have been stored in public databases like SWATHAtlas (https://swathatlas.org). When starting DIA analysis with only a peptide list, a two-step workflow combines spectral reconstruction-based DDA search or sequence-based DIA search with subsequent library-based search can be applied, such as the combination of DIA-Umpire/MSFragger/DIA-Umpire, PECAN/Skyline, or MSFragger-DIA/DIA-NN. These workflows build a library first, followed by targeted peptide extraction and quantification. Alternatively, a library can be predicted from the peptide list and searched against the DIA data in one step.

Additionally, many workflows have been proposed to optimize libraries using the experimental DIA data itself. This involves re-building the library from the DIA data or fine-tuned prediction models to refine potentially unmatched precursor ions or biased peptide attributes. For instance, a library generated based on spectra reconstruction can be used to fine-tune prediction models and refine a library by extending it (153) or smoothly combining two heterogeneous libraries from different sources (154), and the data search reports based on one or multiple libraries can also be combined to generate a self-optimized hybrid library (155, 156).

It is noteworthy that most aforementioned approaches for library generation rely heavily on the accurate prediction of library information. Through algorithm development and leveraging deep learning, predictions of fragmentation patterns (142, 157, 158, 159, 160), RT (161, 162, 163, 164), MS detectability (165, 166, 167), and peptide digestibility (168) have been substantially improved. These are now routinely applied to the identification of modified and unmodified peptides in DIA- and DDA-based proteomics (126, 154, 160, 165, 169, 170). Meanwhile, many tools have been developed to streamline library prediction by integrating multiple steps (142, 154, 160, 165, 171, 172, 173). The topic about library generation through in silico prediction has been extensively overviewed in recent reviews (174, 175, 176, 177).

## DIA Benchmark Datasets

Different combinations of instrument platforms, data acquisition methods and analysis tools have largely increased the versatility of DIA analysis workflows, which in some cases yield inconsistent results of proteome identification and quantification. Therefore, a benchmarking experiment using samples with pre-defined protein/peptide composition and abundance information is critical to assessing the performance of a given DIA analysis workflow. These benchmark datasets are usually acquired from three types of sample designs: type I, synthetic peptide spike-in to a proteome background or solvent; type II, purified protein (*e.g.* UPS1/2 from Sigma consisting of 48 proteins) or peptide mixture spike-in to a proteome background or solvent; type III, multi-species hybrid proteomes. We summarize published DIA benchmark datasets with annotations on the sample design, DIA acquisition scheme and instrument type (Table 6 and online appendix). Almost half of these datasets are generated by developer teams to verify new software packages such as MaxDIA (41) or Specter (102), or new acquisition methods such as diaPASEF (33) or plexDIA (178). The remaining half are mostly obtained by regular users to benchmark and optimize different acquisition methods/platforms, informatic tools, or entire DIA analysis workflows. Concerning the types of proteomics experiments, the majority of benchmark datasets were used in the evaluation of DIA global proteomics, some of which are exemplified in details below. Meanwhile, a small set of datasets was acquired for DIA phosphoproteomics or immunopeptidomics.

**Table 6:** DIA benchmark datasets

Sample composition	Sample type	Instrument	Acquisition methods
422 synthetic heavy-labeled peptides (83)	I	TTOF 5600	Wide-window
UPS1/UPS2 (75)	II	TTOF 5600	Wide-window
UPS1 (179)^a^	II	Orbitrap Fusion	Wide-window/inter-cycle overlapping-window
UPS2 (180)^a^	II	QE HF	Inter-cycle overlapping-window
HYE (181)^a^	III	TTOF 5600+/6600	Wide-window
HYEC (182)	III	QE HF	Wide-window
HY (33)	III	timsTOF Pro	diaPASEF
HYE (41)	III	timsTOF Pro	diaPASEF
HYE (183)^a^	III	TTOF 5600	Wide-window
		TTOF 6600+	Wide-window/scanning SWATH
		Synapt G2-Si	UDMS^E^
		Synapt XS	SONAR
		QE HF-X	Inter-cycle overlapping-window
		timsTOF Pro	diaPASEF
HYE plexDIA (178)	III	QE	Wide-window/MS1-enhanced wide-window
BSA mDIA (184)	II	Orbitrap Exploris 480	Wide-window
HYE mDIA (184)	III	timsTOF HT	diaPASEF/MS1-enhanced diaPASEF
HY (32)	III	ZenoTOF 7600	Wide-window
HE (185)^a^	III	Orbitrap Eclipse	Inter-cycle overlapping-window
MY (186)^a^	III	QE HF/timsTOF Pro	Wide-window/diaPASEF
579 synthetic heavy-labeled phosphopeptides (115)	I	TTOF 5600+	Wide-window
96 synthetic phosphopeptides (102)	I	QE HF Plus	Inter-cycle overlapping-window
200+ synthetic phosphopeptides (135)	I	QE HF-X	Wide-window
166 synthetic phosphopeptides (188)	I	Orbitrap Fusion Lumos	Wide-window
HY phosphoproteome (135)	III	QE HF-X	Wide-window
HY phosphoproteome stoichiometry (135)	III	QE HF-X	Wide-window
Immunopeptides (243)^a^	II	Orbitrap Fusion	Wide-window

All datasets comprise data from tryptic peptides except for the immunopeptide data. H: human; Y: yeast; E: *E. coli*; C: *C. elegans*; M: mouse; BSA: bovine serum albumin.

a From a benchmark study.

Upon the release of OpenSWATH in 2014 (83), the SWATH-MS Gold Standard (SGS) dataset was generated by spiking 422 heavy isotope-labeled peptides into solvent, yeast cell digests, or HeLa cell digests at varying dilution concentrations. SWATH data was acquired on the TripleTOF 5600 instrument using a wide-window scheme. In 2015, three UPS spike-in datasets were obtained on the same instrument using a wide-window scheme along with the introduction of DIA-Umpire (75). In recent benchmarking studies, four datasets were generated for UPS1 spike-in to an *E. coli* cell digest, acquired on Orbitrap Fusion with two fixed wide-window, one inter-cycle overlapping window, and one variable wide-window DIA schemes (179). Additionally, a dataset for UPS2 spike-in to a yeast tryptic digest was acquired on a QE HF instrument with inter-cycle overlapping window scheme (180).

Besides the use of synthetic peptides or purified proteins, a number of benchmark samples are composed of total cell digests from multiple species. A classic benchmark dataset reported by Navarro *et al.* (181) was acquired for hybrid proteome samples comprising human (H), yeast (Y), and *E. coli* (E) digests, referred to as HYE. In one HYE set, sample A comprises H/Y/E at a ratio of 65:30:5, and sample B has a ratio of 65:15:20, which results in defined quantification ratios for peptides from different species. Benchmark data for the HYE samples were acquired on TripleTOF 5600+/6600 instruments using fixed or variable wide-window DIA and analyzed by five different software tools.

Later on, an array of datasets have been generated for hybrid proteome samples such as the HYEC (HYE as defined above and C indicates *C. Elegans*) benchmark acquired on QE HF with variable wide windows (182), HY13 on timsTOF Pro with 16-scan diaPASEF (33), HYE124 on timsTOF Pro (41), and HY12 on ZenoTOF 7600 (32). Notably in 2022, a most comprehensive collection of HYE124 datasets were generated on multiple instruments with specific acquisition methods (183): TripleTOF 5600/6600+ with variable wide-window DIA and scanning SWATH, Synapt G2-Si with UDMS^E^, Synapt XS with SONAR, QE HF-X with inter-cycle overlapping-window DIA, and timsTOF Pro with 16-scan diaPASEF. In addition, Derks *et al.* generated an mTRAQ-labeled HYE dataset to assess isotope labeling-based quantification when they introduced the plexDIA technique for multiplexed quantitative DIA analysis (178). This dataset was acquired on a Q Exactive instrument with both MS1-enhanced wide-window and regular variable wide-window DIA. Later, Thielert *et al.* introduced a dimethyl labelling-based approach, mDIA (184), and generated two datasets for bovine serum albumin (BSA) or HYE samples. These two samples were analyzed on Orbitrap Exploris 480 with wide-window DIA and timsTOF HT with diaPASEF or MS1-enhanced diaPASEF, respectively.

Recently, several benchmark studies investigated how the combination of a software tool and a spectral library impacts the outcome of a DIA analysis workflow implemented on one or multiple instrument platforms (179, 185, 186, 187). For example, an extensive UPS1 spike-in dataset at eight different concentrations was generated by Gotti *et al.* to test 36 DIA workflows (179). A hybrid proteome dataset was created by Fröhlich *et al.* which comprised individual human tissue digests spiked into *E. coli* cell digests to reflect the background heterogeneity of clinical samples (185). This dataset was used to evaluate a high number of DIA data analysis workflows varying in the library generation, software packages, and statistics tools. Lou *et al.* created another hybrid proteome dataset for mouse membrane digests spiked into a yeast proteome background, which were acquired on both Orbitrap and timsTOF instruments (186). This study evaluated 10 data analysis workflows exploiting different DIA software suites and library designs. Later, Zhang *et al.* utilized six benchmark datasets from multiple instruments to assess the performance of DIA software suites operating in a library-based or library-free manner (187). Very recently, Staes *et al.* created an UPS2 spike-in dataset to test 12 different DIA workflows together with one DDA workflow (180).

Beyond global proteomics, a handful of DIA benchmark datasets have been generated for PTM proteomic analysis, especially phosphoproteomics. These include data for synthetic phosphopeptide dilution series acquired on TripleTOF or Orbitrap instruments, allowing for evaluating site localization confidence reported by different analysis tools (102, 115, 135, 188). Moreover, Bekker-Jensen *et al.* created two phosphoproteomics benchmark datasets (135), one from a two-species phosphoproteome sample set in defined ratios, and the other from phosphatase-treated samples to enable phosphorylation stoichiometry determination.

Taken together, various benchmark datasets serve as useful resources for both software/method developers and conventional users to construct, evaluate and improve existing or proposed workflows for DIA-based proteomics.

## Remaining Topics in DIA Data Analysis

With respect to DIA data analysis, this review puts more focus on the analysis strategies and features of currently implemented software tools. For the remaining important topics such as scoring and false discovery rate (FDR) control, PTM analysis, and quantification, we provide a very brief overview here to complement published reviews and include more recent research articles.

A robust scoring system generates discriminative scores for each identified peptide, enabling data analysis pipelines to withstand variabilities in inputs, especially for DIA data where precursors or fragments may be undetectable in libraries. Scoring involves topics such as the sub-score design, the discriminative model, and the training approach. One or more of these concepts have been overviewed previously (9, 83, 189, 190, 191). Beyond scoring, accurate FDR estimation is essential for confident reporting by data analysis pipelines. Important topics here encompass the implementation and theoretical analysis of target-decoy competition (TDC)-based FDR estimation (95, 112, 192, 193, 194, 195, 196, 197), heterogeneous groupwise FDR correction (197, 198, 199), score calibration (200, 201), and so on.

Protein PTM identification remains a focal point in contemporary proteomic and biological studies. Beyond sample preparation (202, 203) and MS acquisition method optimization (31, 66, 135), an ongoing challenge lies in the development of efficient algorithms for accurately localizing PTM sites on peptide sequences. In general, MS data analysis for PTM mapping involves localization probability/score and false localization rate (FLR) estimation (204, 205, 206, 207), arbitrary mass modification detection (208, 209, 210), and diagnostic feature identification (211, 212). Zooming in on DIA, the coelution of modified peptide positional isomers impacts both identification accuracy and site localization stringency. A number of works have reported strategies for confidently and systematically identifying PTM positional isomers in DIA data analysis (115, 118, 135, 213, 214, 215). Additionally, recent review articles provide an overall summary of computational approaches to address specific or multiple PTM types (216, 217, 218).

Accurate quantification inference represents an indispensable module in the entire DIA analysis pipeline, although it is not elaborated on in this review. Currently, the majority of DIA-based quantification approaches rely on peak area integration for assigned precursors and/or fragment ions in a label-free manner. However, key challenges still remain in this process, encompassing, but not limited to, multi-run alignment (117, 219, 220, 221), interference removal or peak correction and selection (57, 84, 119, 222, 223), integration of MS1 and MS2 signals (41, 223, 224), and high-level (*i.e.*, peptide or protein) quantification inference (225, 226, 227, 228). Furthermore, beyond the widely adopted label-free methods, a notable direction is isotope labeling-based quantification for DIA data, which has received growing interest (178, 184, 229, 230, 231, 232). Recent advancements underscore the high accuracy and completeness achieved through labeling-based quantification in conjunction with DIA, particularly in the realm of single-cell proteomic analysis (178, 184). We would expect new reviews dedicated to this important topic that is also intertwined with DIA data acquisition and analysis tools.

## Conclusion and Outlook

DIA has evolved into a next-generation strategy for high-throughput quantitative proteomics. As reviewed here, recent advances in DIA data acquisition schemes and informatic approaches and tools have substantially enhanced the coverage, accuracy and speed of DIA-based proteomics. In regard to instrumentation favoring DIA data acquisition, both scanning quadrupole and ion mobility spectrometry, when coupled with different mass spectrometers, have shown great promises for high-sensitivity and high-speed DIA proteomics. We anticipate future innovations in overlapping-window DIA (including scanning-quadrupole-based) and PASEF-enhanced DIA would further drive DIA towards complete sampling of both the precursor ion and fragment ion beams. Meanwhile, the development of these data acquisition methods would increase data complexity and provoke new challenges to DIA data compression and analysis.

In this review, we classify different DIA software tools based on their analysis strategies. For widely used sequence- and library-based searches, we further designate two approaches to describe how peaks are grouped for scoring. Combining analysis strategies and peak grouping approaches can be complementary to the peptide-centric analysis (94) in specifying workflows implemented in various tools. For instance, among the peptide-centric tools, PECAN combines a sequence-based search with a spectrum-first approach, while OpenSWATH and DIA-NN conduct a library-based search in a chromatogram-first manner. For tools employing a library-based search with a spectrum-first approach, the spectrum-centric MSPLIT-DIA differs from the “combination-centric” Specter. Alternative to the peak grouping step on which we differentiate the sequence-/library-based searches in our review, the workflow specification could be further refined based on the SSM scores, scoring models, and basic scoring units listed in Table 2, Table 3, Table 4, Table 5.

While the repertoire of analysis tools continues expanding, each software package works most efficiently in its own ecosystem. The intermediate and final outputs provided by different software are in distinct formats, complicating integration or re-processing of results from diverse software. Currently, the Skyline ecosystem (233) is the primary platform that can utilize various data sources and integrate results from multiple software packages. Notably, ongoing efforts towards standardizing file formats seek to enhance transparency and flexibility in DIA data analysis (234, 235, 236, 237). For instance, the mzTab format (238) provides well-defined records for identified peptides, scores, PTM localization and confidence, linkages between identifications and MS spectra, etc. This format, supported by ProteomeXchange (239), allows querying peptide identifications from raw MS data, and has been used in software tools like MaxDIA. Furthermore, customization of individual components in a data analysis pipeline rather than treating these tools as black boxes would be critical to establishing an analytical workflow tailored to the DIA data for specific biological systems (240). These joint efforts would ultimately facilitate in-depth mining and retrospective analysis of the vast public DIA proteomic data resources using optimized or updated workflows, which may lead to novel biological discoveries (241, 242).

## Conflict of interest

The authors declare no competing interests.
